# Induction of potent preferential cell death, severe DNA damage and p53-independent ROS-mediated mitochondrial apoptosis by CaTiO_3_NPs in HNO-97 tongue cancer cells

**DOI:** 10.1007/s00210-025-04323-4

**Published:** 2025-06-04

**Authors:** Hanan R. H. Mohamed, Maivel Michael, Yusuf Elberry, Hagar Magdy, Maryam Ismail, Nourhan Eltayeb, Gehan Safwat, Ayman Diab

**Affiliations:** 1 Department of Zoology, Faculty of Science, Cairo University, Giza, Egypt; 2 Faculty of Biotechnology, October University for Modern Sciences and Arts (MSA), 6 Th of October City, Egypt

**Keywords:** CaTiO_3_NPs, HNO-97 tongue cells, SRB assay, ROS generation, Genomic instability, Mitochondrial dysfunction and apoptosis induction

## Abstract

The aggressive nature and high recurrence rate of tongue cancer, along with the severe side effects associated with current conventional treatments, underscore the urgent need for alternative therapeutic strategies that offer improved selectivity and reduced toxicity. Calcium titanate nanoparticles (CaTiO_3_NPs) have previously demonstrated significant cytotoxic effects against MCF-7 breast cancer and A549 non-small cell lung cancer lines. However, their therapeutic potential against tongue cancer remains unexplored. Therefore, this study investigates, for the first time, the cytotoxic effect of CaTiO_3_NPs on HNO-97 tongue cancer cells in comparison to normal human skin fibroblasts (HSF) cells. Using various assays, we assessed their effects on cell viability, reactive oxygen species (ROS) generation, genomic DNA and mitochondrial integrity, and cell death pathways. Cytotoxicity assessment using the sulforhodamine B (SRB) assay demonstrated that CaTiO_3_NPs significantly reduced HNO-97 cell viability in a concentration-dependent manner, with an IC50 of 29.67 µg/ml. In contrast, normal HSF cells exhibited a much higher IC50 of 262.6 µg/ml, indicating strong selectivity for cancer cells (selectivity index = 8.85). Mechanistic studies revealed that HNO-97 cell exposure CaTiO_3_NPs at the IC50 concentration markedly increased ROS production, inducing oxidative stress. This led to significant genomic DNA damage, confirmed by the comet assay, and mitochondrial dysfunction, validated through Rhodamine-123 staining. Flow cytometry analysis indicated the induction of both apoptotic and necrotic pathways. At the molecular level, qRT-PCR analysis revealed significant downregulation of key genes, including the pro-apoptotic *p53*, the anti-apoptotic *Bcl-2*, and the mitochondrial *ND3* gene, supporting the involvement of mitochondrial dysfunction and impaired DNA repair mechanisms in the observed cytotoxic effects. Conclusion: Collectively, these findings demonstrate that CaTiO_3_NPs elicit strong and selective multi-modal cytotoxic effects against HNO-97 tongue cancer cells and minimal impact on normal HSF cells, supporting their promise as a novel nanotherapeutic agent for tongue cancer. Further in vivo studies are needed to validate their therapeutic efficacy, clinical applicability and biosafety.

**Graphical abstract:**

Mechanistic pathway of CaTiO_3_NPs induced cytotoxicity in HON-97 tongue cancer cells

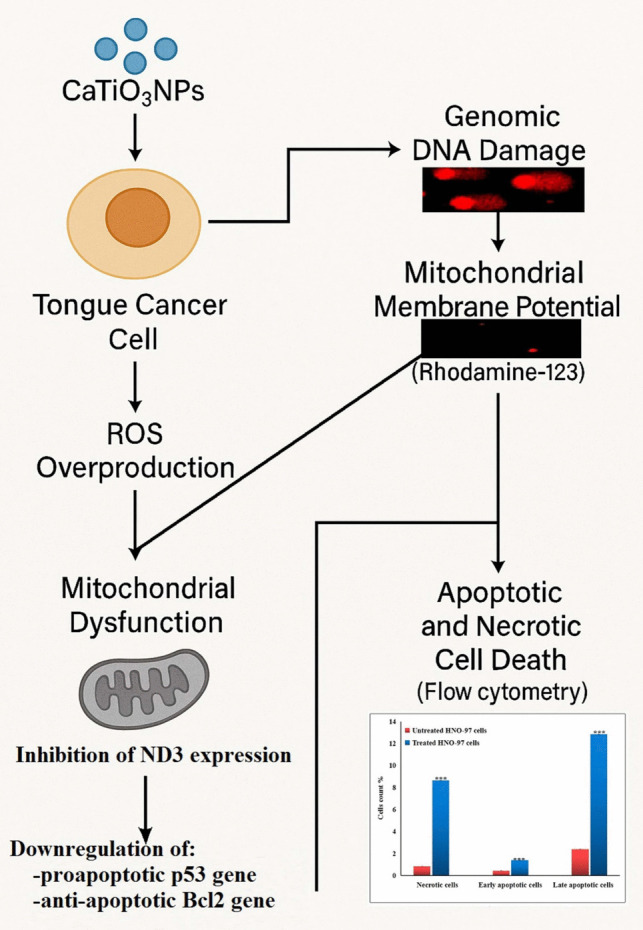

## Introduction

Tongue cancer, a predominant form of oral squamous cell carcinoma, has become an increasingly significant global health concern due to its aggressive nature, rising incidence, and limited therapeutic options. It is the most common malignancy of the oral cavity, accounting for approximately 25–50% of all oral cancers, and is often diagnosed at advanced stages, contributing to high rates of recurrence and metastasis (Warnakulasuriya 2009). Epidemiological studies indicate a noticeable increase in tongue cancer cases, particularly among younger individuals and non-smokers, suggesting shifts in etiological patterns beyond traditional risk factors such as tobacco use, alcohol consumption, and infection with human papillomavirus (Shiboski et al. 2005; Chi et al. 2015). Globally, oral cavity cancers, including tongue cancer, rank among the most common head and neck malignancies, with a notably higher prevalence in males and in regions such as South and Southeast Asia (Shiboski et al. 2005; Bray et al. 2018).

Current treatment modalities for tongue cancer primarily include surgical resection, radiotherapy, and chemotherapy. Among these, chemotherapy remains a cornerstone for managing locally advanced or metastatic disease. However, conventional chemotherapeutic agents such as cisplatin and 5-fluorouracil are often accompanied by severe systemic toxicity, drug resistance, and significant impairment of patients'quality of life (Argiris et al. 2008; Melo-Alvim et al. 2022). Due to their non-specific mechanism of action, these anticancer drugs not only target rapidly dividing cancer cells but also damage healthy tissues, leading to serious complications including mucositis, nephrotoxicity, neuropathy, and myelosuppression (Melo-Alvim et al. 2022; De Felice et al. 2015). These adverse toxic effects of chemotherapy often necessitate dose reductions or early discontinuation of treatment, compromising therapeutic efficacy and patient compliance. Furthermore, the emergence of chemo-resistance remains a major challenge, contributing to disease recurrence and poor long-term survival outcomes (Lustberg et al. 2023). This underscores the pressing need for novel, targeted therapeutic strategies that enhance treatment efficacy while selectively eliminating cancer cells and minimizing systemic toxicity. Nanotherapy has emerged as a promising approach in cancer treatment, offering advantages such as improved drug delivery, tumor-specific targeting, and reduced off-target effects (Peer et al. 2007; Mohamed et al. 2025a).

Among various nanomaterials, calcium titanate nanoparticles (CaTiO_3_NPs) have attracted growing interest due to their notable biocompatibility, chemical stability, and capacity for cellular and subcellular interactions (Bai et al. 2022). In this context, recent research has increasingly focused on the therapeutic potential of CaTiO_3_NPs in cancer treatment. Notably, recent studies by Mohamed et al*.* (2025a, 2022) demonstrated the selective genotoxicity of CaTiO_3_NPs against breast cancer (MCF-7) and non-small cell lung cancer (A-549) cells. These effects were primarily mediated through the overproduction of reactive oxygen species (ROS), which dysregulate apoptotic gene expression, leading to cell cycle arrest and apoptosis in cancer cells, with minimal genotoxicity observed in normal human skin fibroblasts.

Despite these encouraging findings, the therapeutic efficacy of CaTiO_3_NPs has yet to be comprehensively evaluated in tongue cancer, particularly in HNO cell lines. Considering the aggressive behavior of tongue cancer and the limited effectiveness of current treatment modalities, exploring the selective cytotoxicity of CaTiO_3_NPs in HNO cells presents a promising avenue for developing a more targeted and less toxic therapeutic approach. Therefore, this study aims to address this research gap by investigating the effect of CaTiO_3_NPs exposure on the viability of HNO-97 tongue cancer cells and normal human skin fibroblast (HSF) cells. In addition, this study assesses genomic DNA damage, ROS generation, mitochondrial membrane potential disruption, and apoptosis induction in HNO-97 cells to provide a comprehensive understanding of the therapeutic potential of CaTiO_3_NPs in tongue cancer.

Cell viability was determined using the Sulforhodamine B (SRB) assay, while DNA integrity was assessed through the alkaline Comet assay. Furthermore, ROS levels, mitochondrial membrane integrity, and the expression of apoptotic and mitochondrial-related genes were analyzed. Apoptotic cell death was also quantified using flow cytometry.

## Materials and methods

### Chemicals

CaTiO_3_NPs were purchased as white powders (Product No. 633801, 99% trace metals basis) from Sigma-Aldrich Chemical Company (St. Louis, USA). Other chemicals used in this study; dimethyl sulfoxide (DMSO, CAS No. 67–68-5), bovine serum albumin (BSA, Fraction V, ≥ 99%), and Sulforhodamine B (SRB, ≥ 85%), were also sourced from Sigma-Aldrich. Dulbecco’s Modified Eagle Medium (DMEM) with glucose and without phenol red was obtained from Gibco (Thermo Fisher Scientific, Waltham, MA, USA). All other reagents were of analytical or molecular biology grade. For experiments, CaTiO_3_NPs were dispersed in DMSO and ultra-sonicated immediately prior to cell treatment to ensure a uniform suspension.

### CaTiO_3_NPs characterization

**Fig. 1:**
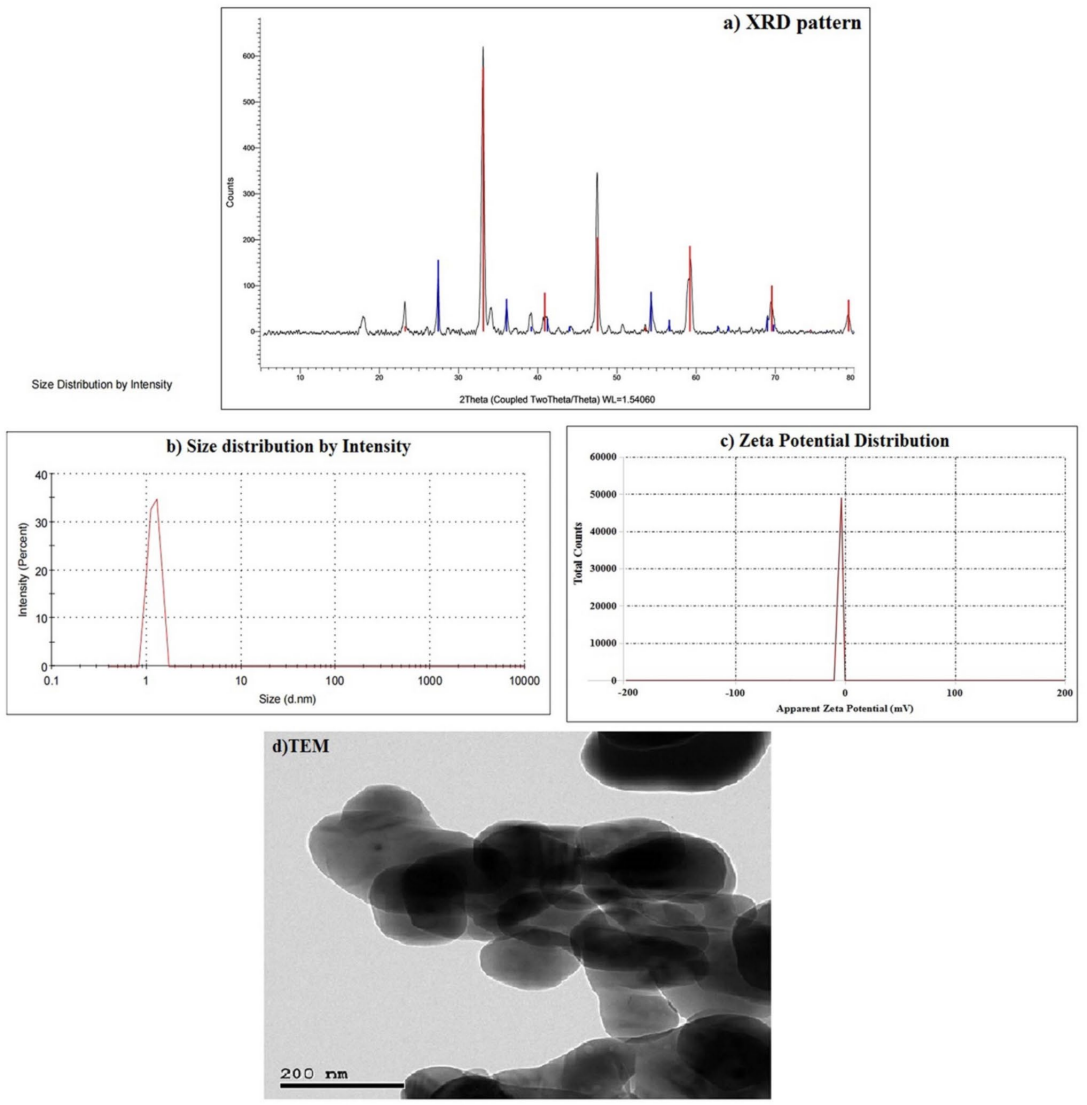
Characterization of CaTiO_3_NPs showing **a**) XRD pattern, **b**) Particle Size distribution, **c**) Zeta Potential distribution and **d**) TEM imaging of CaTiO_3_NPs by Mohamed and Colleagues (2022, 2023)

The CaTiO_3_NPs used in this study were thoroughly characterized in previous work by Mohamed et al*.* (2022, 2023) employing X-ray diffraction (XRD), transmission electron microscopy (TEM), and dynamic light scattering with a Zetasizer Nano Series instrument (Malvern Instruments, Westborough, MA). XRD analysis confirmed the nanoparticles’ high purity and crystalline structure, as evidenced by distinct characteristic diffraction peaks (Fig. 1). TEM images showed predominantly spherical nanoparticles with good dispersion in aqueous media. Furthermore, dynamic light scattering and Zeta potential measurements indicated a nanoscale average particle size of 3.62 nm and a Zeta potential of − 3.38 mV (Fig. 1), demonstrating colloidal stability and minimal aggregation in suspension.

### Cell seeding and culture conditions

Human normal skin fibroblasts (HSF) and HNO-97 tongue squamous carcinoma cells were supplied from Nawah Scientific Inc. (Mokatam, Cairo, Egypt). Both cell types were cultured separately in DMEM supplemented with 10% heat-inactivated fetal bovine serum, 100 Units/ml penicillin, and 100 µg/ml streptomycin. Cultures were maintained under standard conditions in a humidified atmosphere at 37 °C with 5% CO₂ to support optimal cell proliferation and viability.

### Cell viability assessment

The cytotoxic effects of CaTiO_3_NPs on HSF and cancerous HNO-97 tongue cells were evaluated using the SRB assay, following the protocols of Skehan et al*.* (1990) and Allam et al*.* (2018). Cells were seeded at a density of 1 × 10^4^ cells per well in 96-well plates with 100 µl of complete DMEM and incubated for 24 h at 37 °C in a humidified 5% CO_2_ atmosphere. Cells were then treated with five different concentrations of CaTiO_3_NPs (0.1, 1, 10, 100, and 1000 µg/ml) and incubated for 72 h. After treatment, cells were fixed with cold trichloroacetic acid, washed, and stained with 0.4% (w/v) SRB solution for 10 min in the dark at room temperature. Excess dye was removed by washing with 1% acetic acid, and plates were air-dried overnight. The protein-bound dye was solubilized, and absorbance was measured at 540 nm using a FLUOstar Omega microplate reader (BMG LABTECH, Ortenberg, Germany). IC50 values were determined from three independent experiments using GraphPad Prism software. The selectivity index for CaTiO_3_NPs against HNO-97 cells was calculated by dividing the IC50 for HSF by the IC50 for HNO-97 cells.

### Cells treatment

Human HNO-97 tongue cancer cells were cultured in T25 flasks under standard cultured conditions and divided into untreated (control) and treated cells: Control cells were exposed to DMSO at a final concentration of < 0.1%, while treated cells were exposed to CaTiO_3_NPs at the IC50 concentration for 72 h. Following the treatment period, both control and treated HNO-97 cells were trypsinized, centrifuged, and washed twice with ice-cold phosphate-buffered saline (PBS). The collected cell pellets were resuspended in PBS and stored at − 80 °C for subsequent molecular analyses. All treatments were performed in triplicate to ensure reproducibility and statistical reliability.

### Estimation of genomic stability

The alkaline single-cell comet assay was conducted to assess the effect of 72-h CaTiO_3_NPs exposure on genomic DNA stability in HNO-97 tongue cancer cells, following protocols by Tice et al*.* (2000) and Langie et al*.* (2015). A 15 µl suspension containing approximately 10,000 cells was mixed with 60 µl of 0.5% low-melting-point agarose and spread onto slides pre-coated with 1% normal-melting-point agarose. After allowing the gel to solidify for 30 min at room temperature, slides were immersed in cold lysis buffer supplemented with DMSO and Triton X-100 and incubated at 4 °C in the dark for 24 h. Slides were then incubated in alkaline electrophoresis buffer (pH > 12) for 15 min to unwind DNA, followed by electrophoresis at 25 V and 300 mA for 30 min. Post-electrophoresis, slides were neutralized with Tris buffer (pH 7.5) for 5 min, fixed in cold absolute ethanol, air-dried, and stained with ethidium bromide. For each sample, 50 randomly selected cells were analyzed using COMETSCORE™ software. Aberrant comets, including those with overlapping nuclei or excessive background staining, were excluded to ensure data accuracy. DNA damage parameters; tail length, percentage of DNA in the tail, and tail moment, were calculated and expressed as mean ± standard deviation (SD), ensuring statistical robustness.

### Assessment of cellular ROS generation

The effect of CaTiO_3_NPs exposure on ROS generation in HNO-97 tongue cancer cells was assessed using the fluorescent probe 2,7-dichlorofluorescin diacetate (2,7-DCFH-DA), following the method of Siddiqui et al*.* (2010). Equal volumes of HNO-97 cell suspension and 20 µM 2,7-DCFH-DA were mixed, gently agitated, and incubated in the dark at room temperature for 30 min. During this time, the dye entered the cells and was deacetylated by intracellular esterases. In the presence of ROS, the non-fluorescent dichlorofluorescin was oxidized to the highly fluorescent dichlorofluorescein. After incubation, the stained cells were spread as a thin layer on clean glass slides and observed under an epifluorescence microscope at 200 × magnification. Fluorescent images were captured, and ROS levels were quantified by measuring the intensity of green fluorescence using Fiji (ImageJ) software. Fluorescence intensity in CaTiO_3_NPs-treated cells was compared to that of untreated controls to evaluate ROS generation.

### Evaluation of mitochondrial membrane potential

The integrity of mitochondrial membrane potential in HNO-97 tongue cancer cells was evaluated following 72 h-exposure to the IC50 concentration of CaTiO_3_NPs using the fluorescent dye Rhodamine-123, as outlined by Zhang et al., (2011). Equal volumes of Rhodamine-123 fluorescent solution (1.0 µM) and HNO-97 tongue cell suspension were mixed gently and incubated in the dark at 37 °C for 1 h to facilitate dye uptake. After incubation, the cells were washed twice with phosphate-buffered saline (PBS), spread as a thin film on clean, sterile glass slides, and examined under an epifluorescence microscope at 200 × magnification. Fluorescence intensity, reflective of mitochondrial membrane integrity, was captured and quantified using Fiji (ImageJ) software. Comparisons between untreated and CaTiO_3_NPs-treated cells were performed to determine changes in mitochondrial membrane potential.

### Detection of apoptosis and necrosis induction

The induction of apoptosis and necrosis in HNO-97 tongue cancer cells following 72-h exposure to the IC50 concentration of CaTiO_3_NPs was evaluated using dual-channel flow cytometry, in accordance with the manufacturer's protocol for the Annexin V-FITC Apoptosis Detection Kit (Abcam Inc., Cambridge, UK). A dual-laser flow cytometer was employed to differentiate between viable, apoptotic, and necrotic cells. Post-treatment, HNO-97 cells were harvested via trypsinization and washed twice with ice-cold phosphate-buffered saline (pH 7.4). The cells were then incubated in the dark with Annexin V-FITC and propidium iodide (PI) for 30 min at room temperature. Following staining, samples were analyzed using the ACEA Novocyte flow cytometer (ACEA Biosciences Inc., San Diego, CA, USA). FITC and PI fluorescence were detected using the FL1 (λ_ex/em: 488/530 nm) and FL2 (λ_ex/em: 535/617 nm) channels, respectively. A total of 12,000 events per sample were recorded. Data analysis and quantification of apoptotic and necrotic populations were performed using quadrant gating in ACEA NovoExpress software.

### Measurement of *p53*, *ND3* and *Bcl2* gene expression

The mRNA expression level of the pro-apoptotic p53, mitochondrial gene NADH dehydrogenase subunit 3 (ND3), and anti-apoptotic Bcl2 genes were quantitatively assessed in HNO-97 tongue cancer cells following 72-h exposure to the IC50 concentration of CaTiO_3_NPs using quantitative real-time PCR (qRT-PCR). Total RNA was extracted from the untreated and CaTiO₃NPs-treated HNO-97 cells using the GeneJET RNA Purification Kit (Thermo Fisher Scientific, USA), following the manufacturers protocol. Subsequently, 1 µg of purified RNA was reverse transcribed into complementary DNA (cDNA) using the cDNA Reverse Transcription Kit (Applied Biosystems, Foster City, CA, USA). A qRT-PCR was performed for each target gene using SYBR Green PCR Master Mix and gene-specific primers listed in Table 1 (Grzybowska-Szatkowska and Ślaska 2014; Lai et al. 2013; Suzuki et al. 1999). Reactions were run on the StepOnePlus Real-Time PCR System (Applied Biosystems). Gene expression levels were normalized to GAPDH gene expression as a housekeeping reference, and the relative fold changes were calculated using the comparative Ct (ΔΔCt) method. Data are presented as mean ± SD.

**Table 1:** Sequences of primers used in qRT-PCR

Gene	Strand	Primer's sequences
**GAPDH**	**Forward**	**5'-GAAGGTGAAGGTCGGAGTCA-3'**
	**Reverse**	**5'-GAAGATGGTGATGGGATTTC-3'**
**ND3**	**Forward**	**5'-CGCCGCCTGATACTGGCAT-3’**
	**Reverse**	**5'-CTAGTATTCCTAGAAGTGAG-3'**
**BCL-2**	**Forward**	**5'-TCCGATCAGGAAGGCTAGAGT-3'**
	**Reverse**	**5'-TCGGTCTCCTAAAAGCAGGC-3'**
**P53**	**Forward**	**5'-CAGCCAAGTCTGTGACTTGCACGTAC-3'**
	**Reverse**	**5'-CTATGTCGAAAAGTGTTTCTGTCATC-3'**

### Statistical analysis

All data obtained in this study were analyzed using the Statistical Package for the Social Sciences (SPSS). Results are expressed as mean ± standard deviation (SD). Statistical comparisons between CaTiO_3_NP- treated and untreated HNO-97 tongue cancer cells were performed using an unpaired Student’s t-test with Bonferroni correction to adjust for multiple comparisons, ensuring control of the family-wise error rate.

## Results

### CaTiO_3_NPs exhibit strong preferential cytotoxicity toward HNO-97 tongue cancer cells

**Fig. 2:**
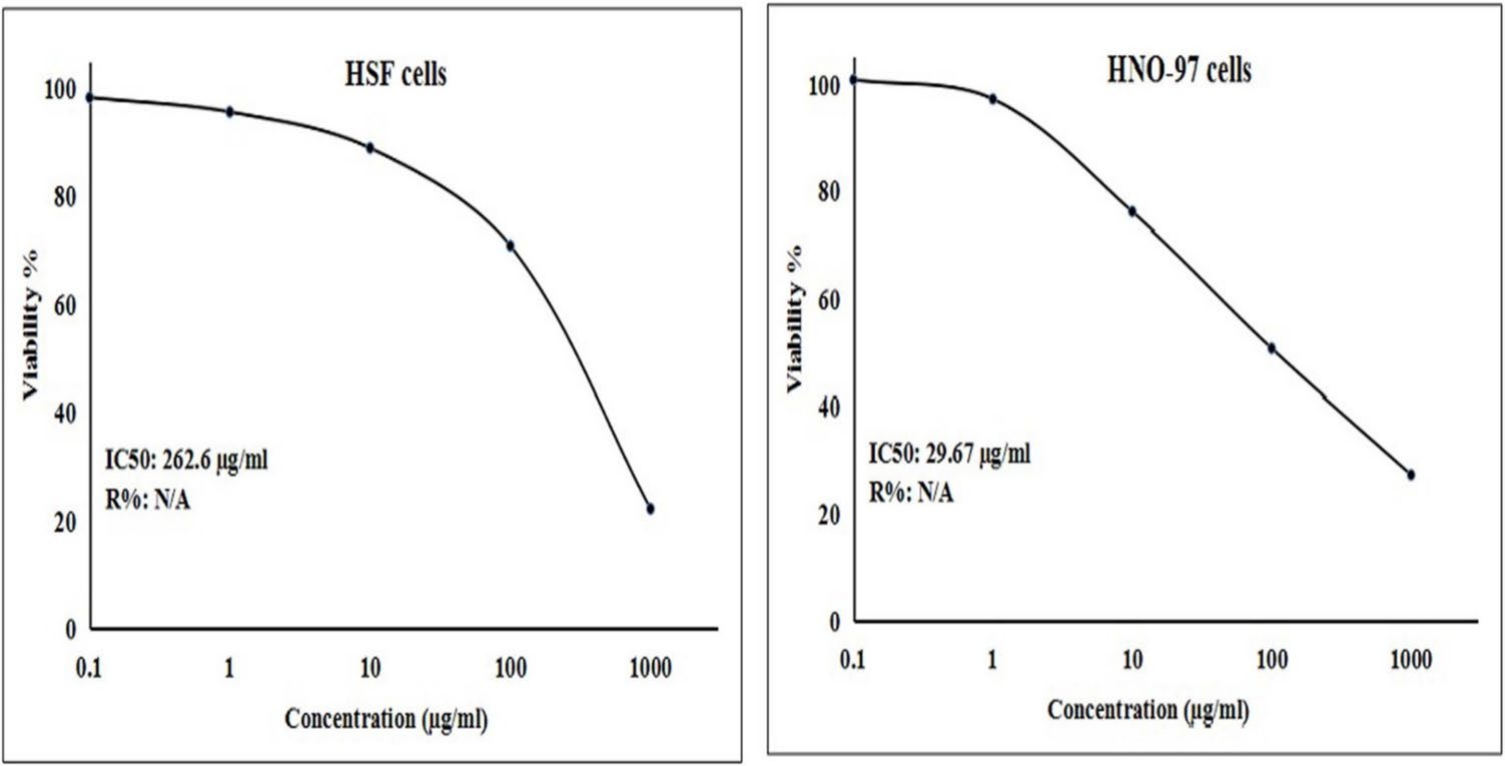
Viability of human normal HSF and tongue cancer HNO-97 cells following exposure to CaTiO_3_NPs various concentrations (0.1, 1, 10, 100 and 1000 µg/ml) for 72 h

Screening the viability of HNO-97 tongue cancer cells using the SRB cytotoxicity assay demonstrated a pronounced, selective cytotoxic effect of CaTiO_3_NPs toward HNO-97 tongue cancer cells. A marked, concentration-dependent decrease in HNO-97 cell viability was observed following 72-h exposure to increasing CaTiO_3_NPs concentrations (0.1, 1, 10, 100, and 1000 µg/ml), yielding an IC50 value of 29.67 µg/ml. In contrast, normal human HSF cells displayed minimal viability loss under the same treatment conditions, with a significantly higher IC50 value of 262.6 µg/ml as shown in Fig. 2. The pronounced selectivity of CaTiO_3_NPs for malignant HNO-97 tongue cells was further demonstrated by a high selectivity index value of 8.85, highlighting their potential as a targeted therapeutic agent for tongue cancer.

### CaTiO_3_NPs severely damage genomic DNA in HNO-97 cells

**Fig. 3:**
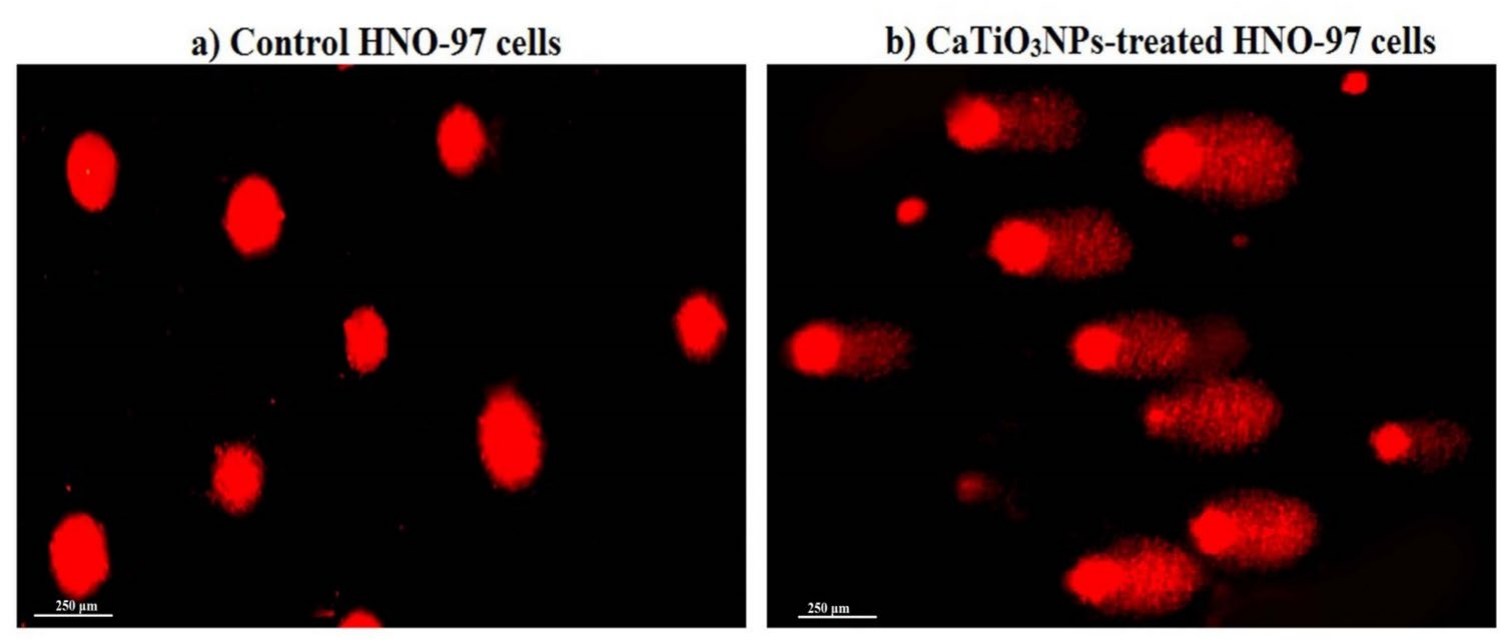
Representative Comet nuclei highlight the contrast between intact Comet nuclei in a0 untreated control cells and those exhibiting pronounced DNA fragmentation in b) HNO-97 cells treated with an IC50 concentration (29.67 µg/ml) of CaTiO_3_NPs for 72 h

Results of the alkaline Comet assay demonstrated a dramatic genomic DNA damage in HNO-97 tongue cancer cells upon 72-h exposure to the IC50 concentration (29.67 µg/ml) of CaTiO_3_NPs. As depicted in Fig. 3 and detailed in Table 2, this damage was evidenced by statistically significant increases (p < 0.001) in DNA damage indicative parameters: tail length, %DNA in tail and tail moment in CaTiO_3_NPs -treated HNO-97 tongue cancer cells compared to their values in untreated control cells (Table 2). Representative Comet images in Fig. 3 further visually highlight the contrast between intact Comet nuclei in untreated cells and those exhibiting pronounced DNA fragmentation in CaTiO_3_NPs -exposed cells.

**Table 2:** Integrity of genomic DNA in HNO-97 Human tongue carcinoma cells following exposure to the IC50 concentration (29.67 µg/ml) of CaTiO_3_NPs for 72 h

Cells	Treatment (µg/ml)	Key DNA damage Comet parameters		
		Tail length (px)	%DNA in tail	Tail moment
**HNO-97 cells**	**CaTiO**_**3**_**NPs (0.00)**	**3.76 ± 1.04**	**18.41 ± 1.43**	**0.69 ± 0.20**
	**CaTiO**_**3**_**NPs (29.67)**	16.65 ± 0.53 ^***^	34.45 ± 1.20 ^***^	5.89 ± 0.13 ^***^

Results are expressed as mean ± SD

***Indicates statistical significant difference from the compared untreated control cells at p < 0.001 using *independent student t-test*

### CaTiO_3_NPs cause excessive ROS generation within HNO-97 cells

**Fig. 4:**
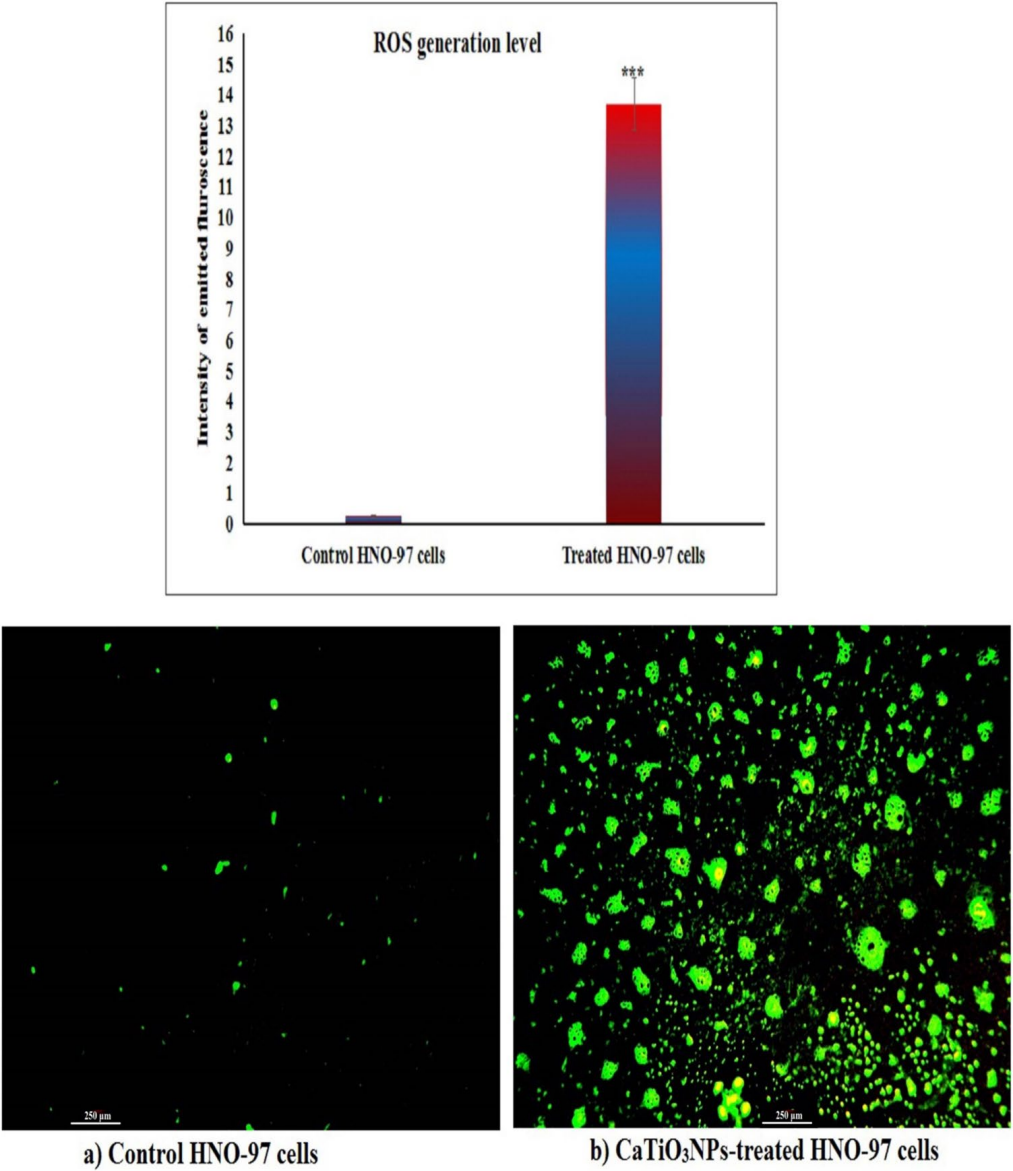
Generation Level of ROS in untreated controls and HNO-97 cancer cells treated with an IC50 concentration (29.67 µg/ml) of CaTiO_3_NPs for 72 h

As shown in Fig. 4, treatment of HNO-97 tongue cancer cells with CaTiO_3_NPs at the IC50 concentration (29.67 µg/ml) for 72 h led to a substantial increase in intracellular ROS generation level. This elevated ROS production was manifested by a significant rise (p < 0.001) in fluorescence intensity in CaTiO_3_NPs-treated HNO-97 tongue cancer cells compared to untreated HNO-97 control cells (Fig. 4), indicating oxidative stress induction by CaTiO_3_NPs exposure.

### CaTiO_3_NPs cause severe loss of mitochondrial membrane integrity in HNO-97 cells

**Fig. 5:**
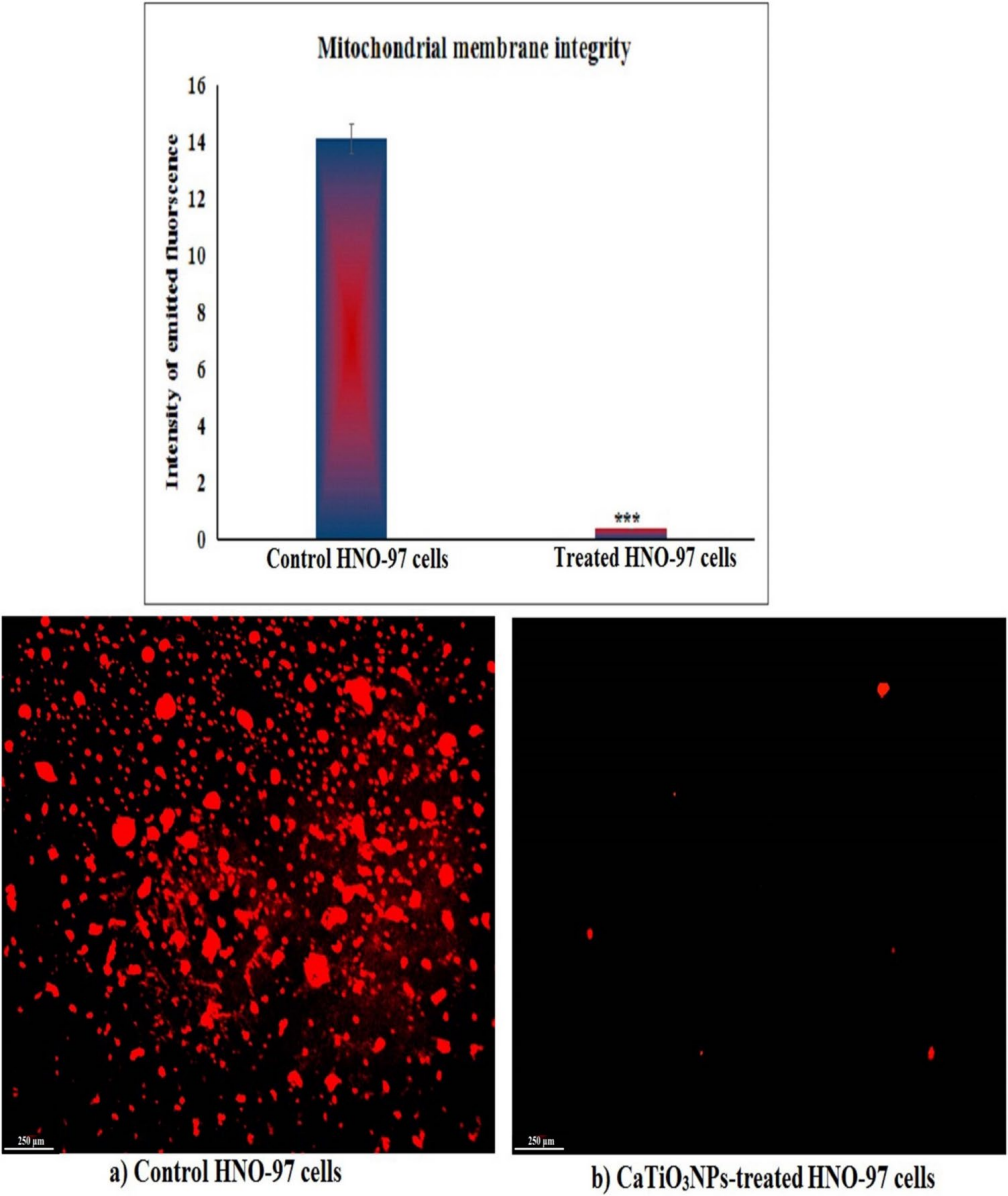
Integrity of mitochondrial membrane potential in untreated controls and HNO-97 cancer cells treated with an IC50 concentration (29.67 µg/ml) of CaTiO_3_NPs for 72 h

Rhodamine-123 staining revealed a marked loss of mitochondrial membrane potential in HNO-97 tongue cancer cells following 72-h exposure to the IC50 concentration (29.67 µg/mL) of CaTiO_3_NPs. As illustrated in Fig. 5, this mitochondrial membrane disruption was indicated by a significant reduction (p < 0.001) in fluorescence intensity emitted by CaTiO_3_NPs-treated HNO-97 tongue cancer cells compared to that emitted by untreated HNO-97 control cells, reflecting severe mitochondrial membrane damage induced by CaTiO_3_NPs exposure (Fig. 5).

### CaTiO_3_NPs promote apoptotic and necrotic cell death of HNO-97 cells

**Fig. 6:**
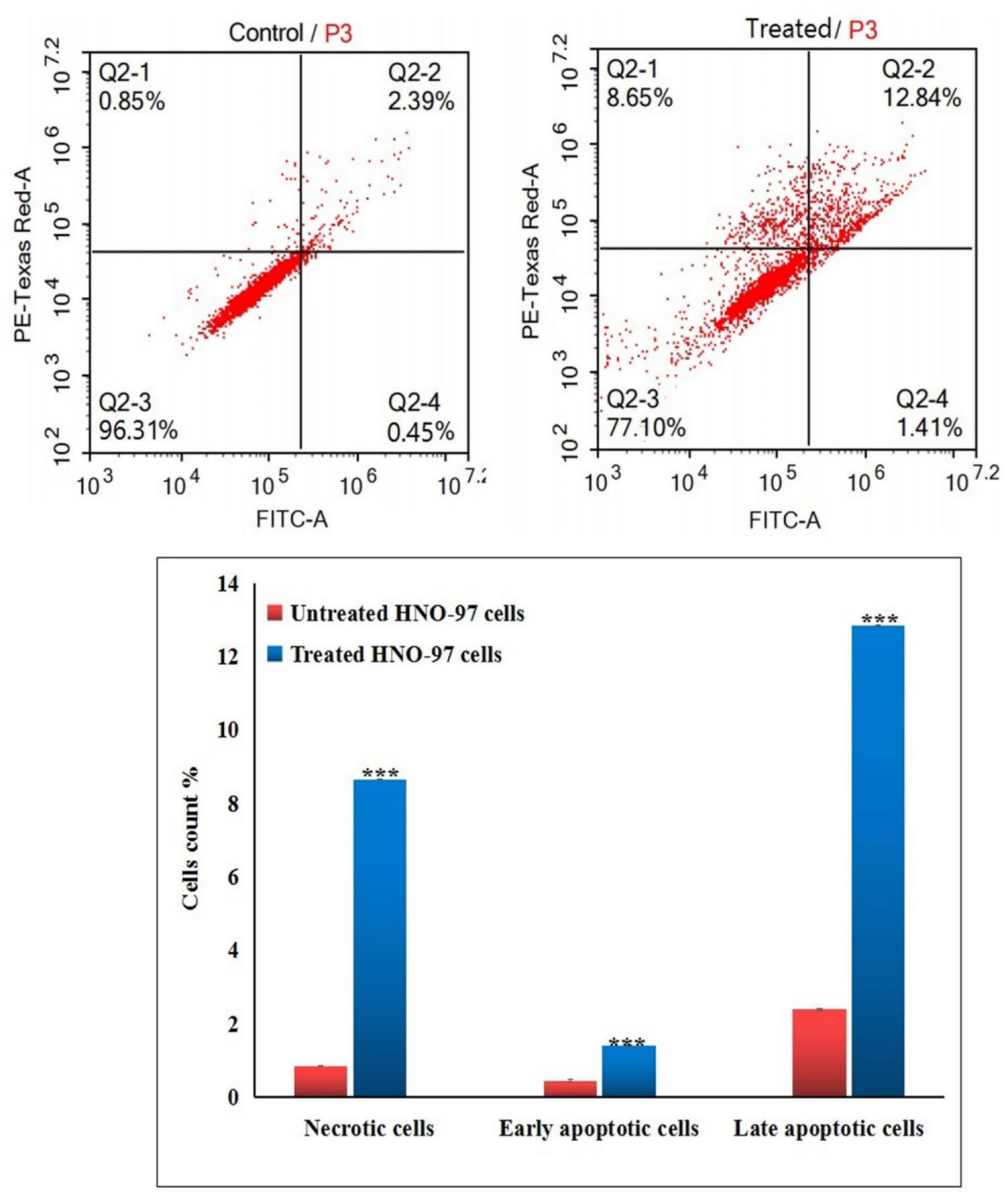
Apoptosis and necrosis induction in untreated controls and HNO-97 cancer cells treated with an IC50 concentration (29.67 µg/ml) of CaTiO_3_NPs for 72 h. Q2-1 denotes necrosis phase; Q2-2 denotes late apoptosis phase, Q2-3 denotes normal viable cells and Q2-4 denotes early apoptosis phase

Discrimination of HNO-97 tongue cancer cells using dual-channel Flow cytometry demonstrated that 72-h exposure to CaTiO_3_NPs at the IC50 concentration (29.67 µg/ml) significantly induced both apoptotic and necrotic cell death in HNO-97 tongue cancer cells. As illustrated in Fig. 6, this effect was detected by a statistically significant increase (p < 0.001) in the proportion of CaTiO_3_NPs-treated HNO-97 cancer cells in early and late apoptosis, as well as in necrosis, compared to the corresponding phases in untreated HNO-97 control cells.

### CaTiO_3_NPs severely dysregulate p53, ND3 and Bcl2 gene expression in HNO-97 cells

Quantitative real-time PCR analysis of gene expression in HNO-97 tongue cancer cells revealed that exposure to the IC50 concentration (29.67 µg/ml) of CaTiO_3_NPs for 72 h resulted in significant dysregulation of key apoptotic, mitochondrial and anti-apoptotic genes. Specifically, treatment with CaTiO_3_NPs led to a marked downregulation (p < 0.001) of the proapoptotic *p53* gene, the mitochondrial *ND3* gene (p < 0.001), and the anti-apoptotic *Bcl2* gene (p < 0.01) compared to their expression level in untreated HNO-97 control cells (Table 3).

**Table 3:** Fold change in the expression level of *p53, ND3* and* Bcl2* genes HNO-97 Human tongue carcinoma cells following exposure to the IC50 concentration (29.67 µg/ml) of CaTiO_3_NPs for 72 h

Cells	Treatment (µg/ml)	Fold change in gene expression		
		p53	ND3	Bcl2
**HNO-97 cells**	**CaTiO**_**3**_**NPs (0.00)**	**1.00 ± 0.00**	**1.00 ± 0.00**	**1.00 ± 0.00**
	**CaTiO**_**3**_**NPs (29.67)**	0.19 ± 0.03 ^***^	0.59 ± 0.06 ^***^	0.81 ± 0.05 ^**^

Results are expressed as mean ± SD

**, ***: Indicates statistical significant difference from the compared untreated control cells at pp < 0.01 and < 0.001, respectively, using *independent student t-test*

## Discussion

Tongue cancer remains a significant health challenge due to its aggressive nature, high recurrence rates, and the substantial morbidity associated with current treatment options. Despite advances in treatment, the therapeutic strategies available for tongue cancer particularly chemotherapy are associated with considerable side effects including nephrotoxicity, hepatotoxicity, ototoxicity, and myelosuppression, highlighting the need for alternative or adjunctive therapies that may offer improved outcomes with reduced toxicity (Vigneswaran and Williams 2014; Driessen et al. 2019).

In this context, nanoparticle-based therapies have garnered increasing interest for their ability to selectively target malignant cells while minimizing collateral damage to normal tissue. Among these, CaTiO_3_NPs have shown promising anticancer potential in *invtro* models such as MCF-7 breast cancer and A549 non-small cell lung cancer, owing to their nanoscale size, high cellular penetration, and favorable biocompatibility (Mohamed et al. 2025a, 2022). These features suggest CaTiO_3_NPs could serve as a novel, targeted approach in cancer treatment. However, the therapeutic potential of CaTiO_3_NPs in tongue cancer remains largely unexamined. Given the unique pathophysiology of tongue cancer and its resistance to conventional treatment, it is critical to explore new avenues of intervention. Therefore, this study was undertaken to evaluate the cytotoxic potential of CaTiO_3_NPs in both normal HSF and cancerous HNO-97 tongue cells. Additionally, the study investigated the CaTiO_3_NPs impact on genomic DNA integrity, mitochondrial membrane potential, ROS production, and apoptosis induction in HNO-97 cells, aiming to uncover their mechanistic role in cancer cell death.

The findings of the SRB cytotoxicity assay demonstrated that CaTiO_3_NPs selectively targeted HNO-97 tongue cancer cells, resulting in a concentration-dependent reduction in cell viability. Notably, exposure to five different concentrations of CaTiO₃NPs (0.1, 1, 10, 100 and 1000 µg/ml) led to a marked decrease in HNO-97 cell viability, with an IC50 value of 29.67 µg/ml. In contrast, normal human HSF cells exhibited only minimal viability loss under the same treatment conditions, reflected by a substantially higher IC50 value of 262.6 µg/ml. This pronounced selectivity is further supported by a calculated selectivity index SI of 8.85, underscoring the potential of CaTiO_3_NPs as a targeted therapeutic agent for tongue cancer. These observations are in line with previous reports by Mohamed and colleagues (Mohamed et al. 2025a, 2022), who also observed strong cytotoxic effects of CaTiO_3_NPs against MCF-7 breast cancer and A549 non-small cell lung cancer cells.

Exploring the molecular mechanisms of CaTiO_3_NPs-induced cytotoxicity is essential to assess their therapeutic potential. Nanoparticles trigger various cellular responses such as ROS production, DNA and mitochondrial damage, and activation of both apoptotic and necrotic pathways, collectively contributing to their anticancer effects (Missaoui et al. 2018; Egbuna et al. 2021).

Accordingly, this study investigated ROS production, genomic DNA damage, mitochondrial membrane disruption, and apoptotic cell death in HNO-97 tongue cancer cells following 72-h exposure to the IC50 concentration of CaTiO_3_NPs aiming to further elucidate the mechanisms driving their selective cytotoxicity.

Initially, a substantial increase in ROS generation was detected in CaTiO_3_NPs-treated HNO-97 tongue cancer cells, as evidenced by a marked elevation in fluorescence intensity following staining with the 2,7-DCFH fluorescent probe. This pronounced ROS overproduction suggests that oxidative stress may be a key mechanism underlying the cytotoxic effects of CaTiO_3_NPs in HNO-97 tongue cancer cells, consistent with previous findings by Mohamed et al. (2025a, 2022), who reported significant ROS generation in MCF-7 breast cancer and A549 non-small lung cancer cells after exposure to CaTiO_3_NPs at their respective IC50 concentrations.

Excessive ROS production can induce oxidative modifications in essential cellular biomolecules, impair mitochondrial function, and activate apoptotic signaling pathways (Hong et al. 2024; Mohamed et al. 2025b). In this study, elevated ROS level correlated with severe genomic DNA damage and significant mitochondrial membrane potential loss. The alkaline comet assay demonstrated that CaTiO_3_NPs significantly increased tail length, %DNA in the tail, and tail moment in HNO-97 cells, confirming extensive DNA fragmentation. Such genomic instability is a hallmark of nanoparticle-induced cytotoxicity and can trigger cell cycle arrest and apoptosis (Mohamed et al. 2025b; Lujan and Sayes 2017). Additionally, the observed loss of mitochondrial membrane potential, indicated by diminished Rhodamine-123 fluorescence, reflects substantial mitochondrial dysfunction. Mitochondrial membrane disruption is a pivotal event leading to the initiation of both apoptotic and necrotic cell death pathways (Mohamed et al. 2025b; Kari et al. 2022).

Induction of apoptotic and necrotic cell death by CaTiO_3_NPs in HNO-97 cells was detected by Flow cytometry analysis through the significant increases observed in the proportion of HNO-97 cells undergoing early and late apoptosis, as well as necrosis, following 72-h exposure to the IC50 concentration of CaTiO_3_NPs in consistent with the findings of Mohamed and colleagues (Mohamed et al. 2022), who reported similar apoptotic and necrotic responses in MCF-7 breast cancer cells treated with CaTiO_3_NPs. These findings suggest that CaTiO_3_NPs not only trigger programmed cell death but also may induce secondary necrosis under sustained stress conditions. Together, the simultaneous activation of multiple cell death pathways likely enhances the overall cytotoxic efficiency of CaTiO_3_NPs against tongue cancer cells.

At the molecular level, qRT-PCR analysis revealed that CaTiO_3_NPs significantly downregulated the expression of several key regulatory genes, including the pro-apoptotic tumor suppressor p53, the mitochondrial gene ND3, and the anti-apoptotic Bcl-2 gene. Unexpectedly, p53 expression was reduced despite its well-known function as a tumor suppressor. This downregulation may indicate cellular exhaustion or the onset of late-stage apoptosis or cellular collapse caused by extensive genomic and mitochondrial damage. Under such severe stress, p53 levels can diminish as cells irreversibly commit to death, aligning with previous studies on p53 behavior during prolonged genotoxic stress (Moulder et al. 2018; Liu et al. 2024; Mohamed et al. 2025c). These findings suggest that the damage induced by CaTiO_3_NPs surpasses the cell’s repair capacity, triggering apoptosis through p53-independent pathways. Without adequate p53 activity, cells are unable to efficiently initiate DNA repair, enforce cell cycle arrest, or activate survival mechanisms in response to DNA damage, resulting in heightened genomic instability and further promotion of apoptosis (Moulder et al. 2018; Liu et al. 2024; Mohamed et al. 2025c). This dysregulation emphasizes the profound cytotoxic impact of CaTiO_3_NPs and underscores the vital role of p53 in preserving cellular integrity under stress.

Moreover, the downregulation of Bcl-2 gene expression following CaTiO_3_NPs treatment likely played a central role in promoting apoptotic cell death in HNO-97 tongue cancer cells. Bcl-2 is a key anti-apoptotic protein that stabilizes mitochondrial membrane integrity by inhibiting the pro-apoptotic proteins Bax and Bak, thus preventing cytochrome c release and the subsequent activation of the caspase cascade (Youle and Strasser 2008). A reduction in Bcl-2 disrupts this protective mechanism, shifting the cellular environment towards apoptosis by allowing mitochondrial outer membrane permeabilization and the activation of intrinsic apoptotic pathways (Kale et al. 2018). Therefore, the significant suppression of Bcl-2 expression observed in this study suggests a collapse of the balance between survival and death signals, strongly favoring apoptotic cell death.

The suppression of ND3 gene expression further underscores mitochondrial dysfunction as a central mechanism of CaTiO_3_NPs-induced cytotoxicity. ND3 gene encodes a critical subunit of mitochondrial Complex I, essential for electron transport, oxidative phosphorylation, and ATP production (Hirst 2013). Its downregulation disrupts Complex I activity and oxidative phosphorylation, leading to diminished ATP synthesis, increased electron leakage, and excessive ROS generation, all of which contribute to mitochondrial instability and oxidative stress (Fassone and Rahman 2012; Zorov et al. 2014). Elevated ROS level can, in turn, intensify damage to mitochondrial and nuclear DNA, proteins, and lipids, amplifying apoptotic signaling and cellular injury (Zorov et al. 2014). As a result, the concurrent downregulation of p53, Bcl-2 and ND3 gene expression disrupts mitochondrial stability at multiple levels by weakening membrane integrity, impairing bioenergetic function and genomic maintenance, thereby promoting robust mitochondrial-mediated apoptosis in CaTiO_3_NPs-treated HNO-97 cancer cells. Collectively, these findings reinforce the notion that targeting mitochondrial pathways represents a promising and increasingly supported anticancer strategy (Fulda et al. 2010; Lebedeva et al. 2009; Lee et al. 2018).

## Conclusion

Overall, the findings of this study indicate that CaTiO_3_NPs exert a potent and selective cytotoxic effect on HNO-97 tongue cancer cells through a multifaceted mechanism involving oxidative stress, severe genomic DNA and mitochondrial damage, and the activation of both apoptotic and necrotic cell death pathways. Notably, this study is among the first to demonstrate the anticancer potential of CaTiO_3_NPs specifically in tongue squamous cell carcinoma, thereby expanding the scope of nanoparticle-based therapeutic strategies. The preferential targeting of cancerous HNO-97 cells over normal cells, alongside with the induction of multiple cell death mechanisms, underscores the therapeutic promise of CaTiO₃NPs in tongue cancer treatment. Despite these findings provide a foundation for further preclinical evaluation of CaTiO₃NPs as novel targeted agents with the capacity to overcome limitations of conventional chemotherapy, further investigation is essential. In vivo studies are needed to validate the efficacy and safety of CaTiO₃NPs in more complex biological environments, where nanoparticle behavior may differ significantly. Additionally, long-term evaluations of genotoxicity, immunogenicity, and biodistribution are critical to ensure safe clinical translation. Future research should also explore surface modification or functionalization approaches to enhance tumor specificity, improve pharmacokinetics, and maximize therapeutic outcomes.

## Data Availability

The datasets used and/or analyzed during the current study are available from the corresponding author on reasonable request.
